# Research Progress on Viruses of *Passiflora edulis*

**DOI:** 10.3390/biology13100839

**Published:** 2024-10-19

**Authors:** Wenhua Wu, Funing Ma, Xiaoyan Zhang, Yuxin Tan, Te Han, Jing Ding, Juyou Wu, Wenting Xing, Bin Wu, Dongmei Huang, Shaoling Zhang, Yi Xu, Shun Song

**Affiliations:** 1 Tropical Crops Genetic Resources Institute, CATAS, National Key Laboratory for Tropical Crop Breeding/Hainan Key Laboratory for Biosafety Monitoring and Molecular Breeding in Off-Season Reproduction Regions, Sanya Research Institute, Germplasm Repository of Passiflora, CATAS, Sanya 571101, China; w2410733376@163.com (W.W.); mafuning@catas.cn (F.M.); qingliangyudi_0621@163.com (X.Z.); tanyuxin1116@163.com (Y.T.); hante221@163.com (T.H.); xingwent@catas.cn (W.X.); wubin@catas.cn (B.W.); huangdm@catas.cn (D.H.); 2 College of Horticulture, Nanjing Agricultural University, Nanjing 210095, China; jding@njau.edu.cn (J.D.); juyouwu@njau.edu.cn (J.W.); nnzsl@njau.edu.cn (S.Z.); 3 Laboratory of Crop Gene Resources and Germplasm Enhancement in Southern China, Ministry of Agriculture and Rual Affairs, Key Laboratory of Tropical Crops Germplasm Resources Genetic Improvement and Innovation of Hainan Province, Haikou 571101, China; 4 Hainan Seed Industry Laboratory, Sanya 572024, China

**Keywords:** *Passiflora edulis*, virus, pathogenesis, control measures

## Abstract

*Passiflora edulis*, also known as passion fruit, is celebrated for its rich nutritional content, distinctive flavour, and significant medicinal benefits. At present, viral diseases pose a major challenge to the passion fruit industry, affecting both the production and quality of the fruit. These diseases impede the sustainable and healthy growth of the passion fruit sector. In recent years, with the expansion of *P. edulis* cultivation areas, virus mutations, and advances in virus detection technology, an increasing number of virus species infecting *P. edulis* have been discovered. To date, more than 40 different virus species have been identified; however, there are different strains within the same virus. This poses a challenge for the control and prevention of *P. edulis* virus disease. Therefore, this review discusses the different types of viruses and their characteristics, modes of transmission, and effects on the growth of the passion fruit plant, as well as the mechanisms of virus generation and preventive measures, with the hope that these discussions will provide a comprehensive understanding of and countermeasures for viruses in passion fruit.

## Simple Summary

Progress in passion fruit virus research has identified infections caused by *Potyvirus*, *Begomovirus* and other viruses, as well as genome sequences of major viruses such as PaVY, TeMV and BYMV, using molecular biology techniques. The transmission, infection cycle, and pathogenesis of these viruses have been elucidated. Virus control strategies include the use of resistant species, RNA interference, physical barriers, and chemotherapy. The focus of this review is on the impact of viruses on fruit quality, the importance of control measures for yield and quality, and the development of more effective prevention and control strategies.

## 1. Introduction

**Figure 1:**
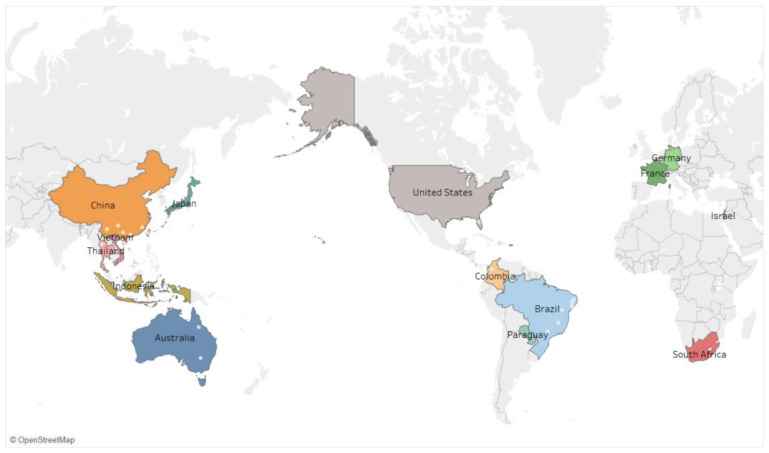
Global distribution map of passion fruit-growing areas. Maps based on longitude (automatically generated) and latitude (automatically generated).

*Passiflora edulis*, commonly known as passion fruit or passionflower, is a perennial vine species belonging to the genus *Passiflora* in the family Passifloraceae. It originates from the north-central region of South America and is classified as a tropical crop [1,2]. It is currently widely cultivated in countries such as Brazil, Colombia, Ecuador, Australia, Vietnam, and China (Figure 1) [3]. According to the Global Biodiversity Information Facility (GBIF), a leading digital repository that houses an extensive collection of biological information accessible via the Internet, there are 1407 species listed within the Passifloraceae family. However, research by Costa et al. suggests that the family comprises approximately 630 species, which are mainly used for ornamental purposes, with only 50–70 species being directly consumable [3,4,5]. Passion fruit is known for its unique flavour, which encompasses the flavours of most fruits. This fruit is also rich in a variety of nutrients, such as amino acids and minerals [5,6]. Research suggests that passion fruit extracts may offer a range of health benefits, such as anti-inflammatory, antioxidant, anticancer, sleep-enhancing, and anti-anxiety properties [6,7,8]. Due to its unique taste and beneficial properties, there is an increasing demand for passion fruit, leading to an expansion of cultivation areas. However, passion fruit cultivation is not without its challenges; it is particularly susceptible to a number of viral diseases that can inhibit growth and development; significantly reduce yields; and, in some cases, cause complete crop failure.

Viral diseases have devastated the area used for passion fruit cultivation in Taiwan, once the leading passion fruit-producing region in Asia [9]. The area shrank from 1392 hectares in 1982, when it was the largest passion fruit production area in Asia, to less than 100 hectares in 1990. Telosma Mosaic Virus (TeMV) has garnered significant attention in recent years and is currently considered to be one of the most serious viruses affecting passion fruit [10]. Passion fruit viral diseases are transmitted by piercing–sucking insects, such as aphids, thrips, and whiteflies [11]. Additionally, industrial propagation of passion fruit often involves methods such as cutting, and the frequent introduction and trade of seeds and seedlings between regions greatly facilitates the spread of these viral diseases. Passion fruit is widely grown in southern Chinese provinces including Guangxi, Yunnan, Guangdong, Guizhou, and Fujian. Recently, reports from those provinces have confirmed the rampant spread of these viruses. An initial survey in regions such as Fujian, Guangxi, Guangdong, and Hainan revealed a virus detection rate of 92% in 112 field samples, demonstrating the prevalence of these viruses in the field [12]. Typical diseases have been found to drastically reduce the expected five-year crop cycle to just one year, with the severity of damage escalating as the area under cultivation expands within the same region [11].

To tackle this challenge, it is crucial to understand the protein structures of different viruses, characterise the diseases they cause and analyse the competitive dynamics between these viruses and the passion fruit plant. At the same time, understanding how viruses are transmitted provides a theoretical basis for blocking their spread. Emerging techniques, such as Agrobacterium-mediated genetic transformation and RNA interference (RNAi), promise to significantly boost the plant’s innate resistance to viruses, offering a more effective and specific alternative to conventional breeding approaches. Furthermore, using physical, chemical, and biological methods to disrupt virus transmission by insect vectors has proven highly effective. Cross-protection strategies, such as attenuated viruses, may also be feasible. Therefore, clarifying the situation of viral diseases in passion fruit and summarising the methods for prevention and control of viral diseases are crucial for the sustainable development of the passion fruit industry.

As an economically important tropical crop, passion fruit is not only unique in flavour but also nutritious and rich in bioactive compounds that have many benefits for human health. However, the prevalence and severity of viral diseases limit the long-term viability of the passion fruit industry. Therefore, investigation of the epidemiology of passion fruit viral diseases, in-depth study of the molecular structure of viruses, exploration of transmission routes, and an understanding of the mechanisms of interaction between viruses and their host plants are essential for the development of effective prevention and control measures. The objective of this study was to elucidate the pathogenetic characteristics of passion fruit viral disease, the mechanism of infection, and the interaction between passion fruit viruses and their host plants. This understanding will provide a scientific basis and technical guidance for the sustainable development of the passion fruit industry.

## 2. Classification of Passion Fruit Viruses

Viral diseases affect the growth and development of passion fruit plants. At present, about 13 genera and more than 40 species of viruses are known to infect passion fruit worldwide (Table 1). Of these, *Potyvirus* and *Begomovirus* are two of the most prevalent genera, comprising 15 and 14 species, respectively. The remaining 11 genera contain a total of 16 species known to infect Passiflora, with viruses such as Cucumber mosaic virus (CMV) and Passion fruit green spot virus (PfGSV) being particularly common.

### 2.1. Potyviruses Infecting Passion Fruit

#### 2.1.1. Proteins Encoded by *Potyviruses*

**Figure 2:**
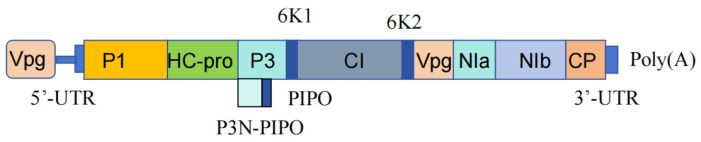
The genome structure of TeMV [52].

*Potyvirus*, within the family Potyviridae, is one of the genera with the widest range of viruses infecting passion fruit. It is the largest genus in the family Potyviridae, and its viruses are primarily spread by aphids through a non-persistent mode of transmission. The Potyviridae family includes viruses with ssRNA polyadenylation genomes encapsulated in curved, filamentous particles, and the *Potyvirus* genome encodes a large polyprotein that, upon proteolytic cleavage, yields 10 mature proteins: P1, HC-Pro, P3, 6K1, CI, 6K2, VPg, NIa, NIb, and CP proteins (Figure 2) [52]. The detailed characteristics of each protein are listed in Table 2 [53,54]. Notably, within this genus, many viruses have an additional short open reading frame, known as PIPO, within the P3 sequence, which is translated by ribosomal frameshifting [55]. The electron microscopy observation of TeMV virus particles in passion fruit sample FJ-13 revealed the linear structure of the virus [56]. These protein structures are also reflected in passion fruit woodiness virus (PWV) [13], Passiflora virus Y (PaVY) [19], cowpea aphid-borne mosaic virus (CABMV) [14], TeMV [23], Ugandan Passiflora virus (UPV) [21], passion fruit chlorosis virus (PaCV) [24], East Asian Passiflora distortion virus (EAPDV) [27], and passion fruit severe mosaic-associated virus (PFMoAV) [26] isolated from passion fruit.

#### 2.1.2. *Potyvirus* Species Infecting Passion Fruit

There are 15 *Potyvirus* species that infect passion fruit, including the major ones such as PWV, CABMV, TeMV, and East Asian Passiflora virus (EAPV) (Table 1). The following genome sequence information is known about the virus strains extracted from passion fruit plants.

The full genetic profile of PWV-Gld-1, discovered in Australia, is 9681 nucleotides long (excluding the poly(A) tail) and contains two open reading frames (ORFs). This virus is a remarkable example of the polymorphic nature of passion fruit *Potyviruses* [13]. The full genome sequence of PaVY, isolated from China, is approximately 9681 nt in length, not including the poly(A) tail, encompassing a substantial ORF of 9252 nt and encoding a polyprotein with a total of 3084 amino acids (aa), with untranslated regions at the 5′ and 3′ ends, measuring 169 nt and 257 nt [19]. The sequence length of CABMV from Brazilian passion fruit is ranges from approximately 9800 to 9930 nt, including two untranslated regions but excluding the poly(A) tail [14]. Bean yellow mosaic virus (BYMV), which can infect a broad spectrum of hosts including legumes and lilies, also affects passion fruit. The ability of the PAC-1 strain to infect passion fruit highlights the adaptability of *Potyviruses* to different hosts. Research suggests that the genetic determinants of BYMV symptoms are located in the N-terminal portion of the CP, a highly variable region among potyviruses, while the 3′ non-coding region (3′-NCR) is critical for host specificity [22,73].

TeMV was first discovered in Vietnam and later found in Thailand, Indonesia, and China. In 2018, the complete RNA genome of TeMV (MG944249) was sequenced in China, comprising 10,049 nt (excluding the poly(A) tail) and encoding a polyprotein of 3173 aa (Figure 2) [23]. UPV-KH7-1, which is up to 9670 nt in length, shares 99.36% homology with UPV isolate UGM-19a (FJ896000) and has a total length of 1718 bp [21]. PaCV has been found in France, Germany, and Israel and was initially identified in 2004 in Florida, USA, where it affected passion fruit plants with chlorosis. The International Committee on Taxonomy of Viruses (ICTV) recognised PaCV as a distinct species of the genus *Potyvirus* in 2008. The complete genome of PaCV spans 9672 bp (excluding the poly(A) tail), and sequence alignment shows the highest similarity to Bean common mosaic necrosis virus (BCMNV) (NC_004047.1) [24]. The soybean mosaic virus (SMV) genome consists of about 9.6 kb of single-stranded, positive-sense, polyadenylated RNA [57].

In recent years, new forms of *Potyvirus* infecting passion fruit have been discovered worldwide, such as EAPDV and PFMoAV. EAPDV, isolated in Japan from 2013 to 2015, consists of 9973 nt and is distantly related to EAPV [27]. In 2019, a new *Potyvirus* virus was discovered infecting passion fruit in Fujian, China, and named passion fruit severe mosaic-associated virus (PFMoAV). Its complete genome sequence is 9974 nt in length (gene ID: MK449340) [25], and it was found to be possibly related to “passion fruit Vietnam potyvirus”, PVNV-DakNong, from Vietnam. A report in 2021 confirmed this discovery, finding that a strain isolated locally in Vietnam (named PaMoV) was identical to PFMoAV [26]. The polyprotein-coding regions of the two viruses exhibit 89% nt and 91% aa consistency, and there is a 99% match in both the nt and aa sequences of their CPs [26].

### 2.2. Begomoviruses Infecting Passion Fruit

The family *Geminiviridae* features a cluster of icosahedral twin plant DNA viruses with circular single-stranded DNA (ssDNA). The criteria for their differentiation include the range of hosts they can infect, the vectors responsible for their transmission, and the organisation of their genomic sequences; on this basis, they can be classified into 14 genera [74,75]. *Begomovirus*, a member of the *Geminiviridae*, is primarily transmitted by the whitefly (*Bemisia tabaci*) [76]. It is also the genus that infects Passiflora.

#### 2.2.1. *Begomovirus* Genome and Encoded Proteins

*Begomovirus* can be further subdivided into monopartite (containing DNA-A) and bipartite (containing DNA-A and DNA-B) types [77]. The monopartite *Begomoviruses*, which include the DNA-A component, are found infecting passion fruit, with the DNA-A playing a crucial role in viral replication, transcriptional activation, and encapsidation. For instance, passion fruit severe leaf distortion virus (PSLDV) contains five open reading frames (ORFs) within its DNA-A, which is one less than the typical number found in *Begomoviruses* [34]. In contrast, Euphorbia leaf curl virus (EuLCV), which also infects passion fruit, encodes seven ORFs within its DNA-A [33]. These ORFs are responsible for the production of various proteins that facilitate infection and pathogenicity (Table 3).

The genetic diversity of *Begomoviruses* infecting passion fruit is enhanced by their ability to undergo recombination and pseudo-recombination events. This genetic plasticity can lead to the emergence of new viral strains capable of infecting a wider range of hosts or causing more severe symptoms in passion fruit. For example, recombination events within the *Begomovirus* population affecting passion fruit have been associated with the emergence of new diseases or the exacerbation of existing ones [85].

#### 2.2.2. *Begomoviruses* Infecting Passion Fruit

*Begomovirus*, a member of the family *Geminiviridae*, is one of the genera that infect passion fruit; members of this genus include passion fruit leaf distortion virus (PLDV) [29], Euphorbia mosaic virus (EuMV) [30], EuLCV, papaya leaf curl Guangdong virus (PaLCuGdV) [31], tomato yellow leaf curl virus (TYLCV) [86], and 14 others (Table 1). Additionally, there are other viruses belonging to the *Geminiviridae* family that can infect passion fruit, such as giant granadilla malformation virus (GGMV) [33], are also capable of infecting passion fruit.

Comparatively, research on *Begomovirus* infections in passion fruit is sparse, especially when compared to the volume of studies on *Potyvirus*. The commercial cultivation of passion fruit is currently less affected by the genus *Begomovirus*, but there is an increasing trend in the severity of the disease as more viruses of this genus are discovered to infect passion fruit. Here, we will list the specific viruses that infect passion fruit and provide information about them. In 2010, a *Begomovirus* called passion fruit severe leaf distortion virus (PSLDV) was discovered in Brazil that can infect passion fruit. Its DNA-A boasts 5 ORFs corresponding to the CP, replication initiator protein (Rep), transcription activator protein (Trap), replication enhancer protein (Ren), and *AC4* genes, while its DNA-B contains two ORFs associated with the movement protein (MP) and nuclear shuttle protein (NSP) genes [34]. Sida mottle Alagoas virus (SiMAV) was discovered to naturally infect passion fruit in 2017 [38]. Passion fruit chlorotic mottle Virus (PCMoV) was discovered in Brazilian passion fruit in 2018, showing a close phylogenetic relationship with both Citrus chlorotic dwarf-associated virus (CCDaV) and Camellia chlorotic dwarf-associated virus (CaCDaV). Due to the large size of the MP they encode (891–921 nt), the genomes of these three viruses are 12–30% larger than those of single-component viruses, and this MP is most closely related to the MP encoded by the DNA-B component of bipartite *Begomoviruses*. Therefore, Fontenele et al. suggest that the viruses belonging to the PCMoV, CCDaV, and CaCDaV lineages may represent a molecular intermediate in the evolutionary step from monopartite *Geminiviruses* (~2.7–3 kb) to bipartite *Geminiviruses* (~5.3 kb) [35].

Moreover, a new virus capable of infecting yellow passion fruit was discovered in Colombia in 2016 and named PLDV [29]. In 2017, EuMV was found to naturally infect passion fruit in Florida, USA; this virus has DNA-A (2609 to 2615 nt) and DNA-B (2571 to 2590 nt) [30].

There are relatively few reports of *Begomoviruses* infecting passion fruit in China, with cases of mixed infections with EuLCV and PaLCuGdV in passion fruit found in Taiwan and Fujian [31,33]. In Yunnan Province, China, passion fruit was found to as a new host of Cotton leaf curl Multan virus (CLCuMuV), a single-component *Begomovirus* with a genome of approximately 2.7 kb and six ORFs [37]. As a monopartite *Geminiviruses*, EuLCV encodes a total of seven ORFs, including AV1 and AV2 encoded on the viral strand and AC1, AC2, AC3, and AC4 encoded on the complementary strand, and the “special” ORF C5, which is also encoded on the complementary strand [33]. Although *Begomoviruses* are transmitted by whiteflies, a virus known as Melochia yellow mosaic virus (MelYMV), which naturally infects passion fruit and cannot be transmitted by whiteflies, has also been discovered in Brazil [36].

### 2.3. Viruses of Other Genera Infecting Passion Fruit

There are 10 other genera and 15 species of viruses that infect Passiflora (Table 1), the most common of which include CMV, PfGSV, and Passiflora latent virus (PLV).

CMV is one of the most common viruses infecting passion fruit due to its wide host range and strong transmission capacity [39]. As a typical member of the genus *Cucumovirus* of the Potyvirus family, CMV is divided into three subgroups, namely, IA, IB, and II [87], with subgroup I predominating in field infections of passion fruit [15]. CMV is an icosahedral, non-enveloped virus, with a triple-component linear positive single-stranded RNA (RNA1, RNA2, RNA3) genome encoding five ORFs, 1a (replicate), 2a, 2b, CP, 3a /MP [88]. RNA1 is responsible for encoding the 1a protein, while RNA2 does the same for the 2a protein. These proteins form the viral replication complex (RC), with 2a containing the RdRp domain and acting as a determinant of symptoms as well [89]. The 2b protein is encoded by the subgenomic RNA4A, which is in turn encoded by the RNA. It is the gene with the most significant variability among the five and has multiple functions as a virulence factor, a suppressor of gene silencing, and a contributor to long-distance viral movement [88,89]. CMV’s CP protein is also encoded by the subgenomic RNA4A [87], and CMV is often accompanied by satellite RNAs [15].

Passion fruit mosaic virus (PafMV) and maracuja mosaic virus (MarMV) are among the *Tobamoviruses* that have been confirmed to infect passion fruit. Viruses of the genus *Tobamovirus* encode at least four proteins in sequence from the 5′ to the 3′ end: an approximately 130 kDa viral replicase protein containing methyltransferase (MT) and RNA helicase (HEL) domains; a 180 kDa viral replicase protein with an RdRp domain; MP, measuring approximately 30 kDa; and CP, measuring about 17 kDa [90,91,92]. PafMV and MarMV are closely related, with their genomic RNA containing around 6700 nucleotides, encoding four ORFs. At the amino acid level, the four ORF sequences of PafMV share 78.8% to 81.6% homology with those of MarMV-P [40,41].

The hibiscus strain of Citrus leprosis virus C2 (CiLV-C2H), from the genus *Cilevirus*, was initially found to infect mainly citrus crops such as oranges and was later discovered to infect legumes and passion fruit. In 2022, passion fruit was discovered to be a new host for CiLV-C2H in Hawaii, USA [43]. PfGSV was previously discovered infecting passion fruit in Brazil in 1997 [42]. The PfGSV genome consists of RNA1 and RNA2 molecules. RNA1 contains two ORFs encoding RdRp and P29 (putative capsid protein). RNA2 contains five ORFs encoding P15, P13/P11, P61, p24, and MP, respectively [93].

Passiflora latent virus (PLV), from the genus *Carlavirus,* infection of passion fruit has been observed in countries such as Germany, Australia, and the United States, and has recently been reported in Korea and China [94,95,96]. The PLV genome found in passion fruit is 8386 nt in length (excluding the poly(A) tail), containing six ORFs with features typical of the *Carlavirus* genus [44,96].

In recent years, Lettuce chlorosis virus (LCV) of *Crinivirus* has also been reported to naturally infect passion fruit, which is found only in the phloem of plants. LCV has a bipartite positive-sense single-stranded RNA genome and is transmitted by whiteflies. Symptoms include mild leaf yellowing, leaf mosaic, leaf deformation, and yellow spots [45].

In 2018, Passion fruit symptomless virus (PeSV), belonging to the genus *Roymovirus*, was isolated from passion fruit plants in Israel. Its genome is 9928 nt long and is most closely related to Rose yellow mosaic virus (RoYMV) [51]. In 2022, a high-throughput sequencing (HTS) survey in Colombia discovered a new virus infecting passion fruit, purple passion fruit leaf distortion virus (PpLDV) of the genus *Tymovirus* in the family *Tymoviridae* with a genome of approximately 6.1 kb in length, closely related to the Perilla mosaic virus (PnMV) [47]. Citrus-associated rhabdovirus (CiaRV) was isolated from passion fruit in China, and the P3 protein of CiaRV shares a common origin with the movement protein of *Begomovirus*, although it belongs to the genus *Rhabdovirus* [49].

Viruses such as Tomato ringspot virus (ToRSV), Passion fruit vein clearing virus (PaVCV), Purple granadilla mosaic virus (PGMV), and PaYMV have been reported to pose less of a risk to passion fruit than viruses such as PVY and CMV, meanwhile having a relatively minor impact on the passion fruit industry. This might be due to several reasons: the Colombian strain of PaYMV and PaVCV may only be transmitted by mechanical means or by grafting [46,48], or they may have a very narrow host range, as is true of PGMV, which only infects Passiflora species [11]; or due to low transmission rates and limited vector ranges, such as the Brazilian strain of PaYMV and PGMV experimentally transmitted by *Diabrotica speciosa* [48,97]; and ToRSV naturally transmitted by *Xiphinema americanum* [11]. Of course, this does not exclude the possibility that there is a lack of large-scale targeted screening.

### 2.4. Evolutionary Relationships of Taxa

**Figure 3:**
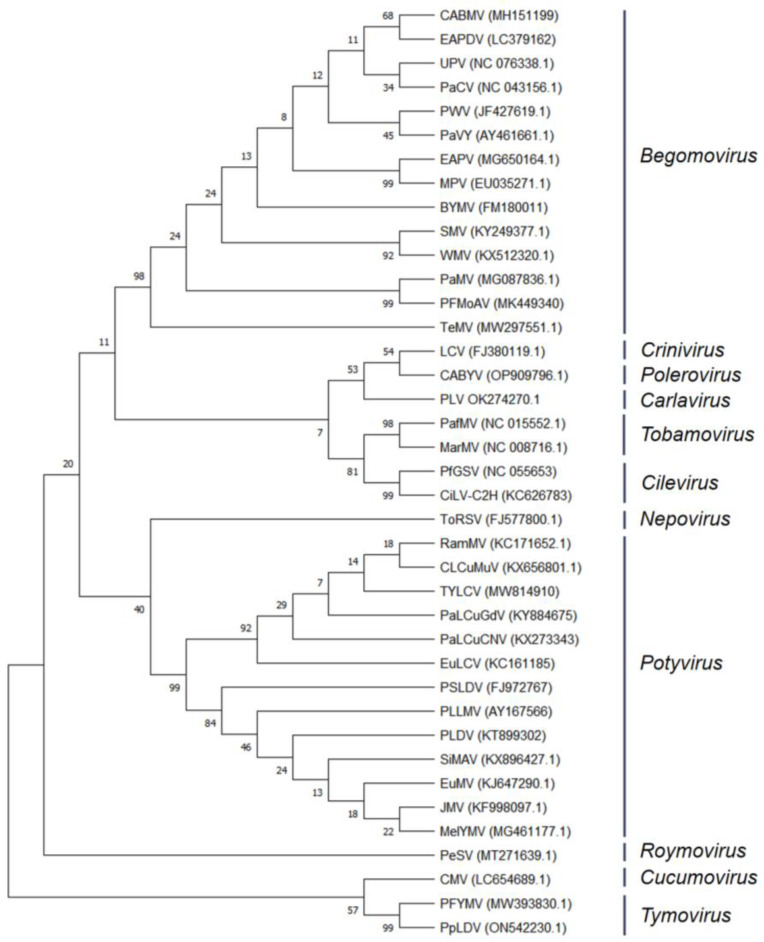
Phylogenetic tree of viruses infecting Passiflora based on nucleotide sequences of the coat protein (CP) gene.

Figure 3 shows the phylogenetic relationships of the viruses found in passion fruit. All data used in this study were obtained from the NCBI GenBank database (https://www.ncbi.nlm.nih.gov, accessed on 1 May 2024), as indicated in Table 1 by the serial numbers, and the sequence analysed was the CP sequence of the virus. The NCBI database and the cited literature contained 39 different species of viruses. The relationship of different strains of *Potyvirus* and *Begomovirus* are explained by this tree. Phylogenetic relationships were established using the Neighbour-Joining method [98]. The resulting optimal tree is illustrated, with branch support values derived from 1000 bootstrap replicates [99]. Phylogenetic distances were determined using the Maximum Composite Likelihood approach [100], which measures the variation in nucleotide substitutions at each site. In this study, a dataset of 28 nucleotide sequences was analysed, including all codon positions along with non-coding segments. Prior to analysis, ambiguous sites were removed using the pairwise deletion technique, resulting in a final dataset with 1135 aligned positions. Evolutionary analysis was performed utilising the MEGA 11 software (www.megasoftware.net, accessed on 1 May 2024) [101].

### 2.5. Viruses Infecting Passion Fruit Around the World

Brazil is one of the largest producers of yellow passion fruit in the world, where CABMV (previously thought to be PWV) is the most widespread and influential virus [102]. *Begomovirus* viruses have also been detected across Brazilian states, such as PCMoV [35], PLLMV [28], PSLDV [34], MelYMV [36], etc., showing a worsening trend [50]. Additionally, PfGSV is widely distributed in Brazil and causes severe damage [42]. In Colombia (Antioquia), the most prevalent viruses infecting purple passion fruit are SMV, PFYMV, CMV, and PpLDV [47]. New viruses spreading in Colombia, such as PfGSV [103] and PLDV [29], have also been found. In Australia, PWV is prevalent [13]. In Vietnam, PWD is primarily caused by PaMoV, followed by EAPV; co-infections with PaMoV and EAPV are common, whereas TeMV infection is rare [26]. In the United States, viruses such as PaCV [24], EuMV [29], PLV [94], and CiLV-C2H [43] have been detected in passion fruit mainly in Florida and Hawaii. In Europe, PaCV and PLV viruses have been reported in France and Germany [24,94].

In China, the predominant viruses vary by region. In Guangxi, 385 samples suspected of viral disease were tested, revealing TeMV, EAPV, and CMV, with severe co-infection phenomena [55]. In Guizhou, the main viruses detected were EAPV, PLV, and TeMV, with detection rates of 63.65%, 34.7%, and 1.59%, respectively [104]. In Guangdong, CMV and PaMV were detected [17]. In Fujian, analysis and identification of suspect diseased samples revealed five viruses infecting passion fruit: CMV, TeMV, EAPV, PaLCuGdV, and PLV [49]. In Yunnan, four viruses (CMV, TeMV, SMV, and PaLCuGdV) were reported to infect passion fruit for the first time [105]. In parts of Hainan, six viruses were detected in 176 samples tested: CMV, CABYV, EAPV, TeMV, PWV, PaMV, and TYLCV [86].

In the future, with advances in genomics and bioinformatics, it is expected that more new viruses that infect passion fruit will be identified. To date, scientists have identified approximately more than 40 viruses that infect passion fruit. Some of these viruses, such as PWV, EAPV, and TeMV, were confirmed early on and are widely distributed in passion fruit-growing areas around the world. In addition, some viruses that originally infected other crops, such as CABMV and CMV, have also become widely distributed in passion fruit-growing areas through various transmission routes. There are also viruses, such as BYMV and ToRSV, that have a relatively small range of spread but affect the healthy growth of passion fruit in various ways and are detrimental to the development of the passion fruit. The structural characteristics of different viruses will help us to understand the properties of different viruses and prepare theoretically for the next steps in virus prevention and control.

## 3. Modes of Transmission of Passion Fruit Viral Diseases

Understanding the modes of transmission of passion fruit viruses is essential for disease management. The main modes of transmission of various passion fruit viruses are by aphids, whiteflies, mites, and other insect vectors that suck plant sap; by grafting and cutting of infected plants; and by mechanical damage caused by pruning and other agricultural practices [64,106]. Viruses such as PWV (*Potyvirus*) are primarily transmitted via non-persistent methods by aphids such as *Myzus persicae*. Aphids can transmit PWV to passion fruit within minutes of acquiring the virus and lose this ability after moulting, which occurs within a short period of time. This mode of transmission is characterised by the rapid spread of the virus without the need for viral replication within the insect, which is typical of PWV in passion fruit [11]. On the other hand, viruses such as PfGSV (*Begomovirus*) are transmitted by the whitefly vector *Bemisia tabaci* in a circulatory, persistent manner. The virus circulates within the whitefly and can be transmitted throughout its life stages, from larvae to adult. The *Begomovirus* CP protein is critical for facilitating intracellular transport of viral DNA, which contributes to efficient transmission [33].

The ability of a virus to replicate within an insect vector differentiates persistent transmission into propagative and non-propagative types. For example, tomato yellow leaf curl virus (TYLCV) can replicate within its whitefly vector, resulting in high transmission efficiency. Studies, such as that by Rosen et al., show that a single whitefly can effectively transmit the virus after an acquisition access period as short as 24 h, with transmission efficiency reaching 100% with a small number of whiteflies [107]. Transmission efficiency can be influenced by several factors, including the biology and behaviour of the vector. Research has shown that artificially feeding whiteflies with anti-HSP70 antibodies can enhance TYLCV transmission, suggesting a role for HSP70 in suppressing begomovirus transmission [107].

Some passion fruit viruses, such as Citrus leprosis virus C2 (CiLV-C2H), are transmitted by the short-palpus mite, *Brevipalpus phoenicis*. This method of circulatory transmission differs from that of whiteflies and aphids, as the virus moves within the body of the mite and is transmitted to the plant [92]. The transmission of viruses such as passion fruit mosaic virus (PfMV) by whiteflies is an example of non-persistent transmission, where the virus does not replicate within the insect and is mechanically transmitted to the plant [11].

Understanding the mechanisms by which the virus spreads is essential for developing effective control measures, which may include controlling insect vector populations or breeding virus-resistant plant varieties to reduce the risk of transmission. In addition, a better understanding of the interaction between viruses and insect vectors may lead to the discovery of new control methods, such as by blocking the process of virus replication or transmission in insects, thereby reducing the damage that the virus causes to plants.

## 4. Biological Characteristics of Viral Diseases Affecting Passion Fruit

**Figure 4:**
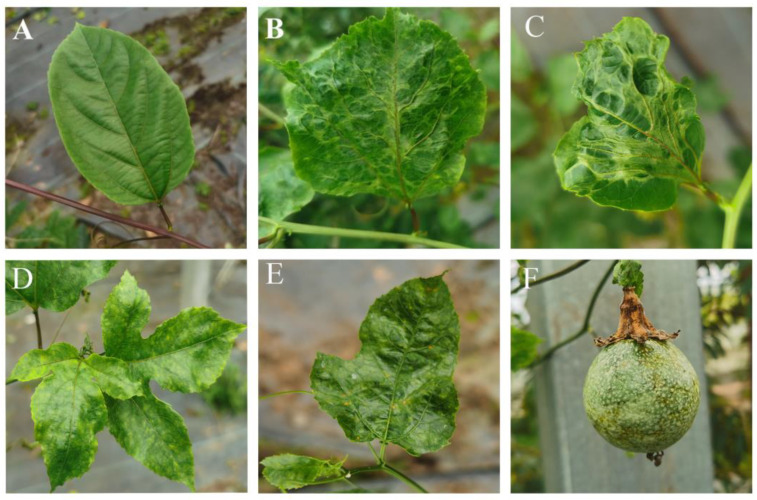
Symptoms of TeMV virus infection in passion fruit. (**A**) Healthy passion fruit leaves. (**B**–**E**) Leaf symptoms after infection with TeMV. (**F**) Fruit after infection with TeMV.

Virus infections are a significant threat to the health and productivity of passion fruit plants. In the leaves, passion fruit virus can cause symptoms such as deformation, wrinkling, mottling, distortion, mosaic, and apical necrosis in affected plants, with older leaves usually showing more obvious symptoms [11,12,23,24,44,108]; some of the symptoms are shown in Figure 4. In the fruit, virus can cause mottling, discolouration, reduced fruit diameter, less fruit flesh, lignification of the fruit, and the appearance of black ring-like spots on the skin after ripening [11,23,24,44,96]. The most detrimental effects of these viral infections are the stunted growth and reduced vigour they cause in the host plants. For instance, PWV can cause severe lignification and stunting in infected plants, leading to significant losses in yield and fruit quality.

Symptoms in passion fruit plants are quite variable, depending on the virus and plant variety. Passion fruit ringspot virus (PFRSV) often causes wrinkling, mottling, and distortion of leaves, with more pronounced symptoms on older leaves [12]. In contrast, EAPV typically causes faded green spots and a mosaic pattern, with fruits from infected plants showing prominent indentations [23].

TeMV and PWV are known to cause severe wrinkling and abnormal stomatal structures when observed under a Scanning Electron Microscope (SEM), indicating their impact on leaf physiology [109]. Certain viruses, such as Watermelon mosaic virus (WMV), show a preference for certain cultivars, primarily infecting purple passion fruit and causing severe wrinkling [24].

Viruses have a significant impact on fruit quality. For example, infection with TeMV significantly reduces the total fat, total acid, and vitamin C content of passion fruit, thereby affecting the nutritional value and marketability of the fruit [57,110].

In some cases, the symptoms are more pronounced on the fruit than on the leaves. PfGSV can cause green spots on the fruits and older leaves and, in severe cases, necrotic lesions around the stems, eventually leading to plant death [42]. It is also important to note that some viruses, such as PFRSV, can cause severe mosaic and deformities on young leaves without causing obvious symptoms on the fruit [11]. This emphasis the need for careful monitoring of both leaves and fruit for early detection of viral infections.

Knowledge of these symptoms is essential for early detection of viral infections. In addition, the virus also affects the nutritional value and marketability of the fruit. Therefore, the study of the interactions between viruses and plant varieties is essential for effective management strategies to reduce the impact of viruses on the passion fruit.

## 5. Plant Immune Mechanisms and the Mechanisms of Disease Symptom Development

Plants, including passion fruit, have evolved a sophisticated array of defence mechanisms to combat viral invasion. These include innate immunity, RNA interference, inhibition of translation, targeted protein degradation via ubiquitination, regulation of gene expression via DNA methylation, protein elimination via autophagy, and the use of key resistance genes [111]. The plant innate immune system is triggered by pattern recognition receptors (PRRs) that identify pathogen-associated molecular patterns (PAMPs) and initiate a series of responses known as pathogen-triggered immunity (PTI). A more specialised response, effector-triggered immunity (ETI), is activated by intracellular nucleotide-binding leucine-rich repeat receptors (NLRs) that recognise specific pathogen effectors [69].

In the context of passion fruit, the interplay between PTI and ETI is critical, with reactive oxygen species (ROS) playing a pivotal role in early signalling events linking these two immune responses [112]. The ETI response, which often involves a hypersensitive response (HR), is more robust and longer-lasting than the PTI. This response is activated when host R gene product containing nucleotide-binding sites and leucine-rich repeats (NBS-LRR) recognise viral effectors, leading to localised cell death that limits viral spread [113].

Pathogenesis-related proteins (PRs), such as PR1, are involved in the plant’s systemic acquired resistance (SAR) and are part of the salicylic acid (SA)-mediated defence signalling pathway, which includes the non-expressor of PR genes (NPR1), a key regulator in the activation of both PTI and ETI [114,115].

The symptoms caused by viral infections in plants are varied and complex. One of the reasons for these symptoms is the plant’s immune response to the virus, in particular the HR and necrotic resistance. When the host R gene product recognises the avirulence factors (Avr) of a pathogen, it triggers necrotic cell death at the infection site, thereby sequestering the virus. This response can lead to changes in levels of defensive hormones, including salicylic acid (SA), jasmonic acid (JA), and nitric oxide (NO), as well as an increase in ROS, which, in turn, activates downstream signalling pathways [111,116].

Moreover, symptoms such as mottling, mosaic, and yellowing are direct indications of changes in the plant’s photosynthetic pigments. Viral infections can lead to reduced photosynthesis, changes in chloroplast structure, and morphological abnormalities. After infection with CMV, chlorophyll content decreases significantly, and a large number of genes related to photosynthesis and chloroplast components are downregulated in passion fruit [39,117]. In passion fruit infected with viruses such as PVY, the rate of photosynthesis is a reduced due to the effect on enzymes involved in chlorophyll synthesis. Chloroplasts, being a preferred target for viruses, often show accumulation of virus replication complexes (VRCs) in their membrane structures, which lack silencing mechanisms [118]. In PVY, the CP protein is also localised to the chloroplasts of the plant; for example, the TeMV-CP protein acts on chloroplasts, causing pathological changes and leading to patchy and mosaic symptoms in leaves infected with TeMV [119].

The immune mechanisms of plants are diverse, but there are few studies on the immune mechanisms of passion fruit. By studying the plant immune system, we will learn more about how plants use intrinsic immune receptors to recognise the mechanisms by which viruses activate defence responses.

## 6. Methods of Controlling Viral Diseases in Passion Fruit

### 6.1. Selecting Disease-Resistant Varieties

As one of the original areas of passion fruit production, Brazil has a rich germplasm resource. In 2021, inoculation experiments with CABMV in 128 genotypes of 12 passion fruit species and three interspecific and intraspecific hybrid types, followed by RT-qPCR verification, indicated that *P. pohlii* and *P. bahiensis* may be immune to CABMV. *P. cincinnata*, *P. gibertii*, *P. miersii*, and *P. mucronata* showed greater resistance than *P. edulis*, *P. alata*, *Passiflora* sp., and hybrids. Varieties that were asymptomatic but carried the virus included *P. suberosa*, *P. malacophylla*, and *P. setacea* [102]. However, most CABMV-resistant species lack commercially viable agronomic traits. Therefore, Sandra C. has opted to use backcrossing to restore the genome of the commercial species (*P. edulis*) [120]. Freitas and others have also shown that intercrossing or backcrossing virus-resistant wild passion fruit resources with commercial varieties results in resistant strains with commendable fruit quality and yield [121,122,123]. In addition to hybrid breeding, artificial virus inoculation can be used to evaluate the field resistance of seedling lines and identify resistant varieties. Studies have shown that yellow passion fruit has greater disease resistance than purple passion fruit [124].

### 6.2. Agrobacterium-Mediated Genetic Transformation

#### 6.2.1. Against Viruses

Using target gene fragments (the virus *CP* gene [125] and *NIb* gene [126]) through Agrobacterium-mediated genetic transformation to introduce them into passion fruit plants and make the transgenic plants resistant to the virus is a common and effective method of molecular breeding. Trevisan et al. used the *CP* gene of the PWV to create transgenic yellow passion fruit plants resistant to the virus, with a genetic transformation efficiency of 0.11% to 0.21% [125]. The *CP* gene transformation of *P*. *alata* with CABMV achieved a genetic efficiency of 0.89% [127]. Silva et al. significantly increased the transformation efficiency in passion fruit somatic embryos (anthers) by ultrasound-assisted Agrobacterium-mediated genetic transformation, with immersion in Agrobacterium suspension for 30 s achieving the highest transformation efficiency of 28.26% [128] New breakthroughs have also been made using seeds as plant material for transformation, where wounds were wrapped with parafilm and plants were kept in the dark for 15 days, achieving a regeneration efficiency of 86% and a transformation efficiency of 29% [129].

#### 6.2.2. Against Vector Insects

Introducing the *Tma12* gene, encoding an insecticidal protein, toxic to the whitefly species *B. tabaci*, from edible fern plants into cotton protected plants from whitefly-transmitted cotton leaf curl virus, while being non-toxic to ladybugs (natural enemies of whiteflies) and rats, indicating minimal ecological impact [130]. Alternatively, the neurotoxin (Hvt) from spiders that prey on insects and the lectin from onion leaves, expressed in the phloem, resulted in almost 100% mortality of whiteflies and had a similar effect on aphids [131]. Since most hemipteran insects, such as whiteflies and aphids, feed on the nutrient-rich phloem, regulating phloem-localised circulation through methods such as controlling phloem proteins (P-proteins) and callose (β-1,3-glucan) to restrict virus movement is an effective strategy. Reducing levels of β-sitosterol (essential for aphid ingestion) to reduce aphid reproduction rates and increasing the accumulation of secondary metabolites in phloem sap to limit aphid feeding and reproduction are other methods [132].

Utilising insecticidal proteins to kill insects that feed on plants is one way to block virus transmission by insects. For the plants themselves, increasing jasmonate levels (jasmonic acid, or JA, and methyl jasmonate, or MeJa) to defend against insect feeding could be another breeding approach. The oxylipin pathway in passion fruit, in which the key member *PfAOS* is derived from AOS-derived jasmonates, is involved in the plant’s defence against pests [133], but studies on this topic are still relatively scarce in passion fruit.

### 6.3. RNAi in Antiviral Applications

RNA interference (RNAi) has the potential to provide broad protection against a number of genetically engineered viruses. In research conducted by Hameed et al. [134], an expression cassette (Ec1/p5941) incorporating the most invariant nucleotide sequences of the coat protein genes of potato virus *X* (PVX), potato virus Y (PVY), and potato virus S (PVS) was constructed to elicit expression of short hairpin RNAs in transgenic potato plants and induce RNAi-mediated resistance [134]. This study established a practical RNAi strategy to achieve broad-spectrum resistance against multiple viruses. As passion fruit often suffers severe losses due to simultaneous infection by multiple viruses, we could consider adopting this strategy to achieve broad-spectrum disease resistance.

Currently, there are still few studies on RNAi technology breeding in passion fruit (no results found in NCBI or through web searches), but some analyses of microRNAs (miRNAs) involved in RNAi have been performed. Paul et al. identified 28 conserved passion fruit miRNAs, belonging to 17 miRNA families, most of which are 21 nucleotides long [135]. The miR166 family had the largest number of members and most of the identified target proteins, including those involved in developmental, metabolic, and defence/stress response signalling pathways such as SQUAMOSA promoter-binding, class III HD-Zip, NAC, Scarecrow, APETALA2, auxin response factors, MYB, and superoxide dismutase, provide a basis for miRNA research in the breeding of disease-resistant passion fruit [135,136].

### 6.4. Physical Control

Physical control focuses mainly on prevention. By understanding the modes of virus transmission, the following measures can be taken: (1) Selection of virus-free seedlings is critical in production; it is essential to select robust seedlings that do not carry viruses. In the commercialisation of passion fruit, propagation by cuttings is predominant, accounting for over 90%, but this method of propagation can lead to varietal degeneration and virus transmission. Therefore, the development of virus elimination techniques for passion fruit is essential. The use of cryotherapy by vitrification for 0.8~1.0mm shoot tips of passion fruit has been proven effective, with a virus elimination rate of 100%, and the highest survival and regeneration rates of 83.3% and 60%, respectively [119]. (2) Timely removal of infection sources in the field, such as annual clearing of orchards to remove weeds and dead branches, thus reducing the breeding of pests and diseases. (3) Not planting near crops of different families such as Leguminosae, Cucurbitaceae, Solanaceae, etc. (4) Employing measures such as insect-proof barriers, nets, and traps to control intermediate hosts of the virus. (5) Ensuring that pruning tools are disinfected to prevent mechanical transmission of the virus.

### 6.5. Chemical Control of Viruses

In the past, various insecticides, such as neonicotinoids, organophosphates, organochlorines, carbamates, pyrethroids, abamectin, and spiromesifen, have been used against whiteflies and aphids [137,138]. For controlling the Passion fruit green spot virus, acaricides such as hexythiazox and fenpyroximate have proven effective [11]. However, insects are increasingly developing resistance to both traditional and novel compounds. In 2010, whitefly populations from south-eastern China were found to have high levels of resistance to pyrethroids and neonicotinoids (such as imidacloprid and thiamethoxam) [139]. A 2018 report indicated that the MED cryptic species of Chinese whitefly has developed resistance to cyantraniliprole [140]. Long-term and excessive use can not only lead to resistance in insects but also potentially harm the environment. It is therefore advisable to select new environmentally friendly insecticides. The PA1b peptide (pea albumin 1, subunit b) extracted from legume seeds has shown potential as a plant-derived insecticide and has been shown to have insecticidal activity against some aphids and is harmless to humans and mammals [141].

Furthermore, choosing agents that directly inhibit viruses and using Plant Resistance Inducers (PRIs), also known as Plant Immunity Agents, is also a good approach [137,139]. In terms of viral inhibitors, agents such as validamycin∙morantel (Jiangxi Heyi Chemical Co., Ltd., Jiujiang, China), morantel hydrochloride (Henan Siyuan Biotechnology Co., Ltd., Luohe, China), and a 4% solution of xinaomycin, a nucleoside antibiotic (Henan Jiyuan Baiyun Industrial Co., Ltd., Jiyuan, China), were ideal for controlling passion fruit flower mosaic virus [142]. In the case of high disease index, the control effect was still more than 57% after 30 days of application. In addition, 20% Morantel hydrochloride has been shown to be effective against CMV-infected passion fruit by significantly reducing the expression of the CMV *CP* gene, and upregulation of disease-resistance related genes [117]. Polysaccharide PRIs, such as chitosan (CS) and phosphate cross-linked chitosan (PCS), extracted from fungal cell walls and arthropod exoskeletons, inhibit CMV and enhance the expression of defence-related enzymes [143,144].

In antiviral research, apart from chitosan and β-glucan, other agents such as amino-oligosaccharide [117], lentinan (LNT), and sulfated lentinan (sLNT) [145], Ningnanmycin [146], dufulin (an α-aminophosphonate), and cytosine peptoid are also being investigated for inducing plant immunity [147]. Ferulic acid derivatives, chalcones, pentadienone, quinazolinone, ketone, and vanillin derivatives are also explored as antiviral agents [148].

### 6.6. Mild Strain Cross-Protection

Utilising attenuated strains to inoculate plants can induce specific antibodies in the plants, protecting them from more severe damage caused by the same virus. This method is known as Mild Strain Cross-Protection (MSCP). The key to the production of attenuated strains in the genus *Potyvirus* is their HC-Pro. Using the four conserved motifs of EAPV-TW HC-Pro, double mutants of EAPV (EAPV _I8_N_397_ and I_181_N_397_) were constructed that effectively controlled EAPV strains in Taiwan and Vietnam [149]. In the studying of PaMoV virus strains, single or double amino acid mutations were also made in the N-terminal conserved motif of HC-Pro, ultimately finding that the RNA silencing suppression (RSS) of PaMoV-E_53_I_181_ was inhibited, providing good protection for passion fruit [150]. In researching attenuated CMV strains, specific amino acid positions of the 2b protein are often mutated. The 2b protein of CMV is a key determinant for attenuating toxicity, a multifunctional protein that suppresses RNA silencing and counteracts salicylic acid (SA)-mediated host basal resistance [151,152]. Wang et al. also generated attenuated TeMV mutants by inducing selective mutations at key positions in the conserved FRNK motif of HC-Pro [153]. These mutants were capable of pervasively infecting the health of passion fruit plants without causing apparent symptoms and effectively prevented subsequent infection by virulent TeMV strains.

However, MSCP has its drawbacks. In the last century, pioneering research in Australia using mild strains for cross-protection effectively controlled PWV, but it was reported that as more severe strains emerged, they could overcome the protection of the mild strains and, in combination with CMV infection, lead to synergistic effects causing apical necrosis disease and other problems [11].

The results of this research will not only improve the efficiency of disease management in passion fruit but also identify more anti-virus passion fruit species. By inoculating attenuated strains and the controlling novel pathogens, the impact of the virus on the passion fruit can be reduced, protecting growers’ economic interests and providing consumers with healthier and safer food choices. In addition, as molecular biology technology advances, there may be more gene editing solutions for specific viruses in the future to enhance the natural resistance of passion fruit.

## 7. Conclusions

In recent years, significant progress has been made in understanding passion fruit viruses, including virus classification, modes of transmission, infection cycles, and prevention methods. Scientists have used molecular biology to analyse the genome sequences of viruses such as PaVY, TeMV, and BYMV in the genus *Potyvirus*, and GGMV, PSLDV, and PLDV in the genus *Begomovirus*. These studies have revealed the genetic structure of the viruses and the interaction between the viruses and plants. However, prevention remains a challenge due to virus variability and multiple modes of transmission, including insect vectors. Future research will focus on analysing the genetic structure and pathogenic mechanism of the virus; exploring efficient, environmentally friendly control methods such as RNAi technology and nanoparticle delivery; and strengthening interdisciplinary collaboration. Recognising the complexity and long-term nature of prevention, comprehensive and multi-level strategies need to be implemented, taking into account environmental sustainability. Although progress has been made, the challenges remain significant and will require continued basic research, exploration of new treatments, interdisciplinary cooperation, and environmental protection for the sustainable development of the passion fruit industry.

## Figures and Tables

**Table 1:** List of the virus species that infect Passiflora.

Genus	Name	Abbreviation	GenBank	Symptoms of Virus-Infected Passion Fruit	References
Potyviridae, *Potyvirus* (PVY)	Passion fruit woodiness virus	PWV	JF427619.1	Leaf twisting, lignification of fruits, and smaller fruit.	[13]
	Cowpea aphid-borne mosaic virus	CABMV	MH151199	Leaf wrinkled and twisted, with greenish, ringed spots; fruit lignified and malformed; slow plant development.	[14]
	Passion fruit ringspot virus	PFRSV	--	Mottled and ringed spots; severe leaf deformation; fruit asymptomatic.	[15,16]
	Passion fruit mottle virus	PaMV	MG087836.1	Leaves lightly mottled; fruit epidermis mottled.	[17]
	Soybean mosaic virus	SMV	KY249377.1	Leaves mottled, wrinkled, and deformed.	[18]
	Passiflora virus Y	PaVY	AY461661.1	Yellow-green mottling, ring spots, faded green spots, and curled leaves.	[19]
	East Asian Passiflora virus	EAPV	MG650164.1	Vein necrosis and rugosity of the upper trifoliate leaves; misshapen, woody and pitted fruit; stunted vegetative growth.	[20]
	Ugandan Passiflora virus	UPV	NC_076338.1	Foliar mosaics, vein clearing, fruit hardening, and malformation	[21]
	Malaysian Passiflora virus	MPV	EU035271.1	-	[12]
	Bean yellow mosaic virus	BYMV	FM180011	Deformed leaves with mosaic.	[22]
	Telosma mosaic virus	TeMV	MW297551.1	Deformation of flowers, leaves, and leaflets; fading green leaves with mosaic; small fruits; uneven colouring of fruits.	[23]
	Watermelon mosaic virus	WMV	KX512320.1	Mosaic and severe leaf crumpling.	[24]
	Passiflora chlorosis virus	PaCV	NC_043156.1	Chlorotic.	[24]
	Passion fruit severe mottle-associated virus	PFMoAV or PaMoV	MK449340	Severe foliar mosaic; stunted growth; mottling, yellowing, and distortion of leaves; small, woody, and twisted fruits.	[25,26]
	East Asian Passiflora distortion virus	EAPDV	LC379162	Mosaic and curled leaves; fruit deformed.	[27]
Geminiviridae, *Begomovirus*	Passion fruit little leaf mosaic virus	PLLMV	AY167566	Severely yellowed and greatly reduced foliage; small, mostly misshapen fruits; fewer fruits set on a single plant; drastic reduction in foliage layer and plant growth.	[28]
	Passion fruit leaf distortion virus	PLDV	KT899302	Yellow foliage and leaf deformation.	[29]
	Euphorbia mosaic virus	EuMV	KJ647290.1	Mottled yellowing, distortion, and apical necrosis of leaves; bright foliar mosaic begins with light mottling, followed by necrotic spots, leaf distortion, and flower abortion.	[30]
	Euphorbia leaf curl virus	EuLCV	KC161185	Systematically mottled and malformed leaves; yellowing, twisting, and necrosis at the top of the leaves; striped concave surfaces on the surface of immature fruits.	[31]
	Papaya leaf curl Guangdong virus	PaLCuGdV	KY884675	Mosaic patterning, mottling, yellowing, crumpling, and twisting of leaves.	[31]
	Passion fruit leaf mottle virus	PLMV	--	Severe curling, twisting, and mottling of leaves and fruits.	[15]
	Ramie mosaic virus	RamMV	KC171652.1	Stunting, mosaic, and yellow or necrotic spots.	[32]
	Tomato yellow leaf curl virus	TYLCV	MW814910		[15]
	Papaya leaf curl China virus	PaLCuCNV	KX273343.		[15,33]
	Passion fruit severe leaf distortion virus	PSLDV	FJ972767	Dwarfing, leaf twisting, and greenish coloration.	[34]
	Passion fruit chlorotic mottle virus	PCMoV	NC_040706.1	Chlorosis, wrinkling, and leaf distortion.	[35]
	Melochia yellow mosaic virus	MelYMV	MG461177.1	Mosaic, yellow spots, and leaf curling and deformities.	[36]
	Cotton leaf curl Multan virus	CLCuMuV	KX656801.1	Leaf curling and vein swelling.	[37]
	Sida mottle Alagoas virus	SiMAV	KX896427.1	Severe mosaic with yellow spots, leaf deformities, and blisters.	[38]
*Geminiviridae*, other genus	Giant granadilla malformation virus	GGMV	--		[33]
*Cucumovirus*	Cucumber mosaic virus	CMV	LC654689.1	Mosaic and yellow spots on leaves; severely curled, raised, and whitened epidermis of fruit.	[39]
*Tobamovirus*	Passion fruit mosaic virus	PafMV	NC_015552.1		[40]
	Maracuja mosaic virus	MarMV	NC_008716.1	Mosaics or mottling; necrotic spots.	[41]
*Cilevirus*	Passion fruit green spot virus	PfGSV	NC_055653	Leaves mottled, faded green spots; yellow spots on senescent leaves with green bands of veins; green spots on fruit and older leaves; and, in severe cases, deadly necrotic lesions around the stems.	[42]
	Hibiscus strain of Citrus Leprosis Virus C2	CiLV-C2H	KC626783	Green spots on young leaves.	[43]
*Carlavirus*	Passiflora latent virus	PLV	OK274270.1	Inconspicuous systemic mosaic; senescent leaves mottled; faded green spots; systemic faded green necrosis of leaves and mottling of upper leaves; and black annular blotches on the surface of ripe fruit.	[44]
*Crinivirus*	Lettuce chlorosis virus	LCV	FJ380119.1	Slight yellowing, mosaic, leaf distortion, and yellow spots.	[45]
*Tymovirus*	Passion fruit yellow mosaic virus	PFYMV	MW393830.1	Mosaic, vein mottling, wilting, and leaflet deformation.	[46]
	Purple passion fruit leaf deformation virus	PpLDV	ON542230.1	Leaf curling, leaf distortion, and ruffling.	[47]
*Nepovirus*	Tomato ringspot virus	ToRSV	FJ577800.1		[40]
*Rhabdoviridae*	Passion fruit vein clearing virus	PaVCV	--	Reduction in leaf area and fruit size in addition to bright veins on the leaves.	[48]
	Purple granadilla mosaic virus	PGMV	--	Mildly linear leaves; small, deformed, and woody fruits.	[11]
	Citrus-associated rhabdovirus	CiaRV	--	Yellow and green spots.	[49]
*Polerovirus*	Cucurbit aphid-borne yellows virus	CABYV	OP909796.1	Wrinkling, mosaic, leaf and fruit deformation, blistering, yellow spots, vein whitening, purple leaves, yellowing and thickening of old leaves, and reduction in fruit number.	[50]
*Roymovirus*	Passiflora edulis symptomless virus	PeSV	MT271639.1		[51]

**Table 2:** Types of proteins encoded by the Potyvirus genome.

Name	Protein Description	Action Mechanism	Related Research
P1-Protease	Serine protease	The first (N-terminal) mature protein of all monopartite viruses, which is highly polymorphic and the most variable and least conserved region in the genome, plays a role in influencing intercellular virus spread and determining host range.	P1 can enhance the activity of HC-Pro in the genus *Potyvirus*, interfering with host defence mechanisms and inducing the production of HSP70 heat shock proteins [57].
Helper component-proteinase, HC-Pro	Rich in cysteine protease motifs, it is essential for aphid transmission	KLSC and PTK MOBS in HC-Pro and DAG MOBS in CP can promote aphid transmission of SMV [58].	HC-Pro can inhibit host plant gene silencing by binding to double-stranded RNA (dsRNAs) and suppressing Dicer processing and the accumulation of 21-nucleotide short interfering RNA (siRNA) [59]. HC-Pro also plays a significant role in reducing photosynthetic rates after PVY infection in plants [60].
Cylindrical inclusion, CI	CI protein possesses ATP binding and RNA helicase activities, as well as NTPase activity, and is also a component of the viral replication complex	CI protein is involved in viral replication through its helicase domain and C-terminal region. The N-terminal sequence is associated with intercellular movement. It may assist in virus genome replication by binding to RNA through its helicase domain and C-terminal region, thereby unwinding RNA double strands.	Likely involved in intercellular movement through the formation of cone structures on plasmodesmata (PD) and interaction with the capsid protein (CP) [61].
P3-Protease	Transcriptional slippage at a single-nucleotide insertion site within the P3 cistron generates an additional peptide, P3N-PIPO	Regulates viral replication, movement, and pathogenesis.	P3N-PIPO localises to plasmodesmata (PD), interacts with the CI protein, and facilitates intercellular movement of the virus in susceptible hosts [62]. P3 is a determinant of virulence in soybean mosaic virus (SMV) [63].
6K1	Located at the periphery of infected cells, rich in hydrophobic amino acids, and associated with membrane binding	6K1 may be involved in intercellular movement [58,64].	6K1 inhibits JA-dependent defence and suppresses aphid reproduction [65].
VPg	It plays a crucial role in the translation or replication of positive-strand RNA viruses, serving as an intrinsically disordered protein, a characteristic that endows it with the ability to bind to multiple proteins	VPg exists in various precursor forms, such as 6K2-VPg-NIa-Pro, which is recruited into the viral replication complex; VPg-Pro-Pol serves as a primer for replication; VPg covalently binds to the 5′ end of RNA, serving as a determinant of virulence [66,67].	VPg interacts with the eukaryotic translation initiation factor 4E (eIF4E), playing a crucial role in virus RNA replication [66,68].
Nuclear inclusion b-protease, NIb	An RNA-dependent RNA polymerase responsible for replicating the viral genome	Recruited into the Viral Replication Complex (VRC) through interaction with the VPg domain of 6K2-VPg-Pro.	NIb is also crucial for the formation of the viral replication complexes (VRCs) and is involved in multiple virus-host interactions. For example, NIb acts as an inhibitor of host defence responses [69] and engages in an arms-race-like antagonism with NPR1 (Nonexpresser of Pathogenesis-Related Genes 1, which is a major regulatory factor in salicylic acid-mediated plant local and systemic acquired resistance) [70].
Nuclear Inclusion a-protease, NIa-Pro	A cysteine protease with trypsin-like activity associated with the small ribonucleic acid virus 3C proteinase	The small ribonucleic acid virus 3C protease can cleave hundreds of host proteins to facilitate viral infection [71], and NIb is released by the NIa protease [69].	NIa often exists in stable intermediate forms, such as the previously mentioned 6K2-VPg-NIa-Pro [67,71]. NIa also participates in RNA replication, interacting with viral RNA-dependent RNA polymerase (RdRp) and viral RNA to stimulate viral RNA replication [71].
Coat protein, CP	The main structural protein of the virion	CP (Coat Protein) has a conserved DAG motif near the N-terminus of the protein, which is involved in the interaction between CP and HCPro, associated with aphid transmission [72]. The coat protein is also involved in virus replication, movement, symptom expression, RNA encapsidation, and other processes [23,72].	Usually plays a role in the production and spread of symptoms [54].

**Table 3:** *Begomovirus* protein species encoded by the genome.

	Name	Protein Description	Action Mechanism and Related Research
DNA-A	AV1/CP/V1	Coat protein	As the CP protein, it is also involved in the intracellular transport of viral DNA and transmission by insects.
	AV2/MP/V2	Movement proteins, not present in the bipartite *Begomoviruses* [77]	AV2 is a potent suppressor of Post-Transcriptional Gene Silencing (PTGS) and Transcriptional Gene Silencing (TGS), mediating the nuclear export of CP [78,79].
	AC1/Rep/C1	Replication initiator protein	AC1 is crucial for replication and may play a key role in the recruitment and assembly of the viral replication mechanisms.
	AC2/TrAP/C2	Transcription activator protein	AC2 is a pathogenic factor that suppresses host defences and is also a gene silencing suppressor, interfering with the ubiquitination pathway and jasmonic acid signalling [80].
	AC3/Ren/C3	Replication enhancer protein	C3 can enhance viral replication, increasing the amount of virus accumulated in the host [79].
	AC4/C4	Multifunctional protein, an inhibitor of RNA silencing.	C4 is a determinant of symptoms and one of the main means of plant defence, with the amino acid sequence of this protein showing the greatest variability. It is involved in the suppression of RNA silencing (PTGS and TGS) and has the ability to disrupt JA (jasmonic acid) signalling [74]. Recent research has found that the C4 protein also participates in regulating the severity of leaf curling during symptom development [81].
	AC5/C5	Present in some monopartite viruses, it is a determinant of virulence	It can suppress transcriptional gene silencing induced by single-stranded RNA, aiding in viral infection [82,83,84].
DNA-B	BV1/NSP	Nuclear shuttle protein	Involved in the development of symptoms [76].
	BC1/MP	Movement protein	Involved in viral movement [76].
	Other small ORFs, of which the largest is named V3.	Located in the Golgi apparatus, it acts as an RNA silencing suppressor	It is essential for complete viral infection [78].

## Data Availability

No new data were created or analyzed in this study.
